# A Case of Low T1 Mapping Values in Myocardial Calcifications in a Patient With End-Stage Renal Disease

**DOI:** 10.7759/cureus.82431

**Published:** 2025-04-17

**Authors:** Ahmed Abdelmonem, Ahmed S Negm, Subhi Al'Aref, Kedar Jambhekar

**Affiliations:** 1 Radiology, University of Arkansas for Medical Sciences, Little Rock, USA; 2 Cardiology, University of Arkansas for Medical Sciences, Little Rock, USA

**Keywords:** cardiac mri, diffuse myocardial calcifications, end-stage renal disease (esrd), t1 mapping, tissue characterization

## Abstract

Cardiac MRI is the gold standard in diagnosing many cardiac pathologies. T1 mapping, a novel cardiac MRI sequence, provides precise assessment and characterization of myocardial tissue. Low T1 values have been reported in specific entities, including Fabry disease and iron overload.

We report a case of decreased T1 values with diffuse myocardial calcifications in a 24-year-old female with end-stage renal disease. This case provides valuable new insights into T1 mapping, particularly regarding the association between myocardial calcifications and low T1 values, a connection that has only been reported in a single prior study.

## Introduction

Cardiac MRI is an invaluable tool in evaluating myocardial diseases [1]. T1 mapping is a relatively newer sequence that can help diagnose certain myocardial pathologies. This is performed by measuring the tissue spin-lattice or longitudinal relaxation time and presenting it on a parametric map. This sequence has attracted considerable attention [2,3].

Elevated T1 values have been instrumental in the diagnosis of conditions such as myocardial fibrosis, amyloidosis, and myocarditis. Conversely, low T1 values have been linked to very few conditions such as iron deposition disease, Fabry disease, and fat deposition [4-9].

We present the case of a young female with end-stage renal disease (ESRD) who underwent a cardiac MRI to evaluate for myocarditis. Incidental note was made of low T1 values, which corresponded to diffuse myocardial calcifications when compared with a non-contrast chest CT. To our knowledge, myocardial calcifications have only been previously reported once as a cause of low T1 mapping values in a patient with acute myelogenous leukemia. Furthermore, ESRD is generally associated with elevated myocardial T1 values due to an increased incidence of myocardial fibrosis [10]. However, in our case, the findings were contrary to this expectation.

## Case presentation

**Figure 1:**
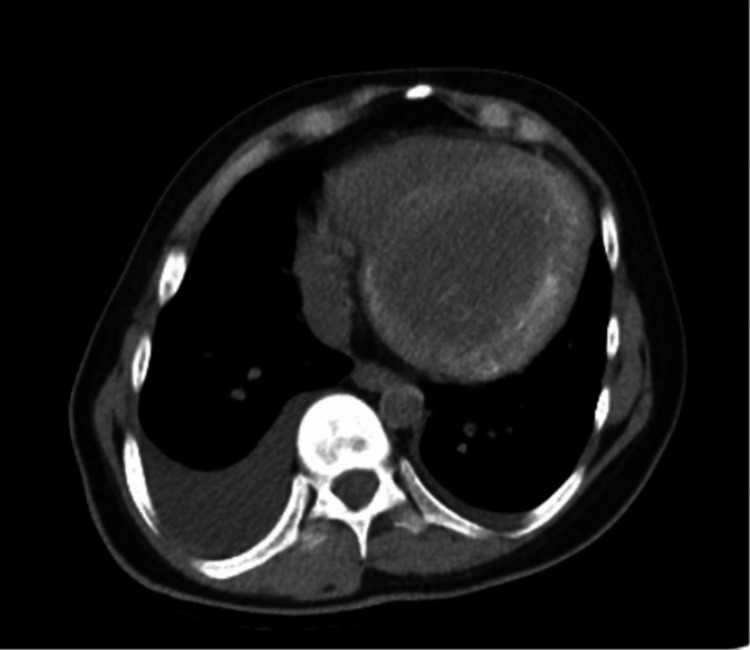
Non-contrast axial CT of the chest showing significant cardiomegaly with diffuse hyperdense calcifications in the left ventricular myocardium. Bilateral pleural effusions (more profound on the right) are also seen.

**Figure 2:**
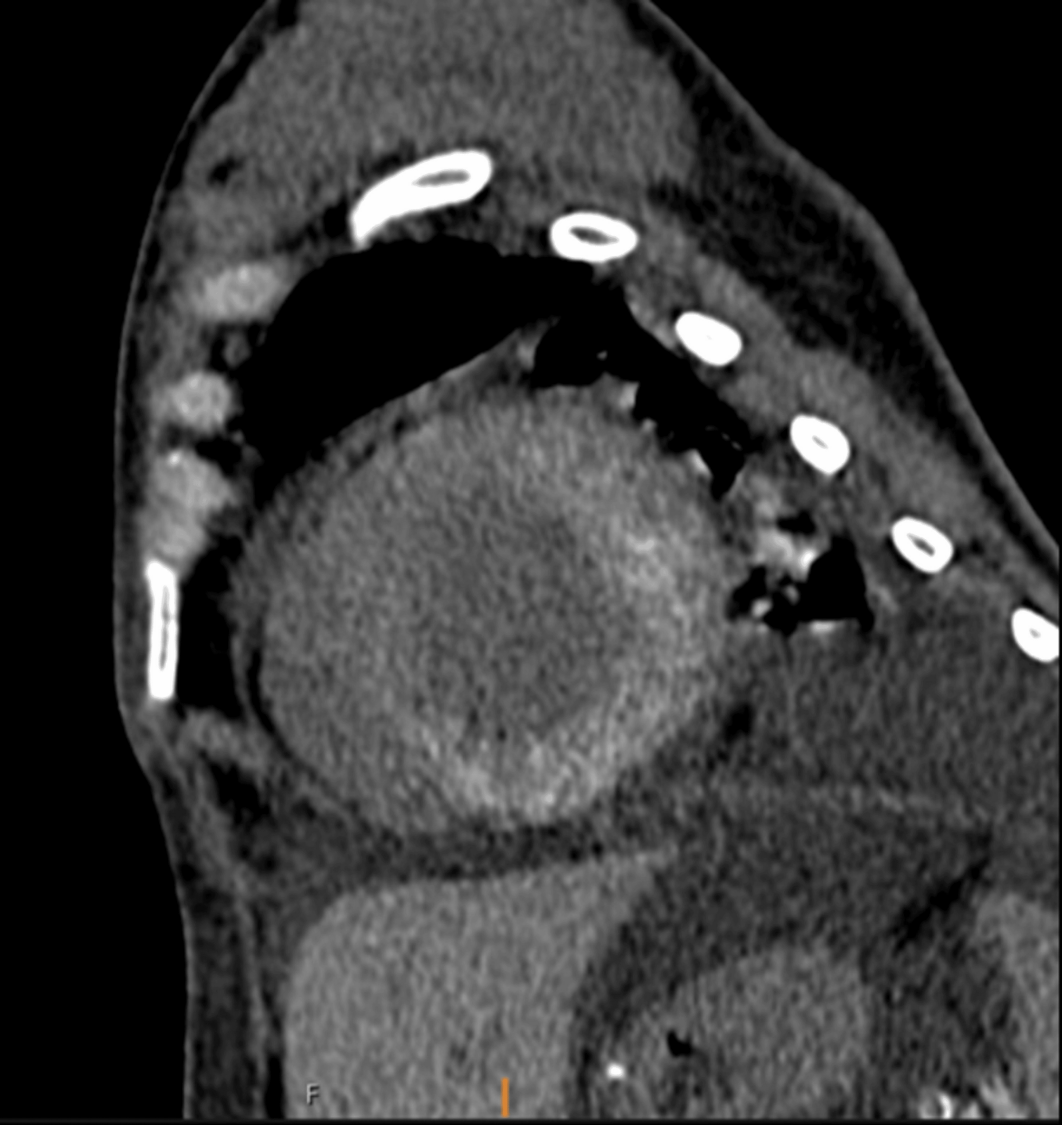
Cardiac CT with short-axis views showing diffuse hyperdense calcifications in the left ventricular myocardium. Pleural effusion is also seen.

**Figure 3:**
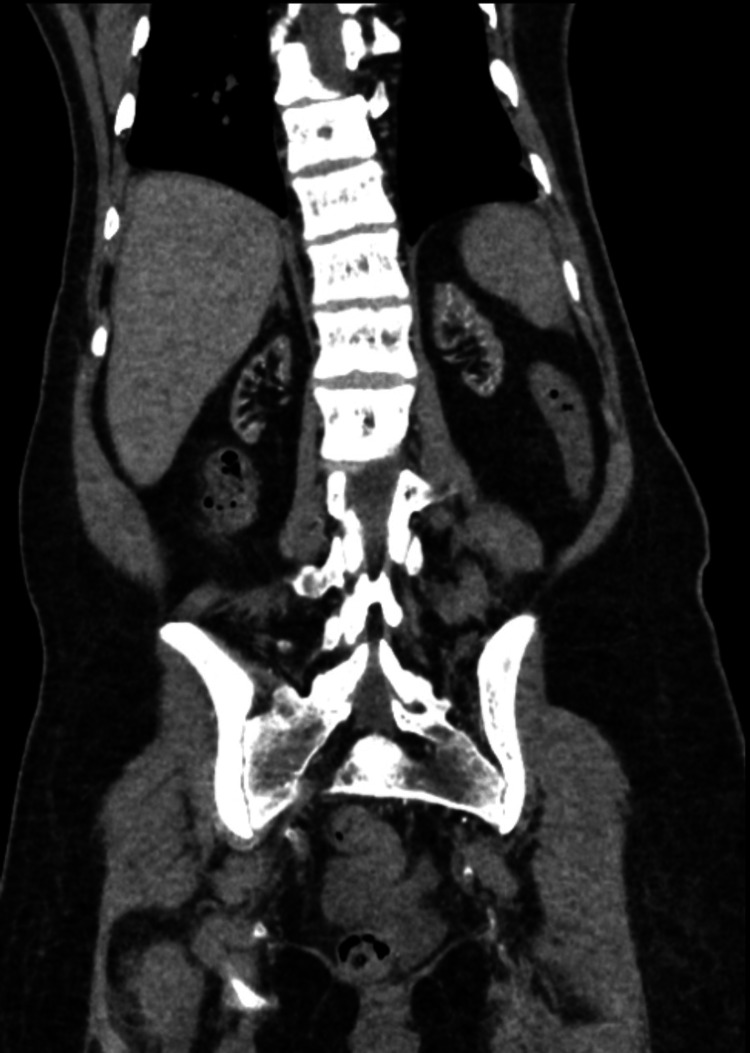
CT of the abdomen and pelvis demonstrating atrophic kidneys, consistent with the patient’s diagnosis of end-stage renal disease.

A 24-year-old female with ESRD who was undergoing evaluation for renal transplantation presented with acute shortness of breath and hypoxemic respiratory failure secondary to pulmonary edema in ESRD. Echocardiography revealed a decreased ejection fraction from 35-40% to 20%. A cardiac MRI was performed for suspected myocarditis. Contrast was not administered due to the patient’s renal status. T1 maps were conducted to evaluate the myocardium, which revealed decreased T1 times. On reviewing earlier CTs (Figures 1, 2), it was noted that the patient had diffuse myocardial calcifications related to her ESRD (Figure 3).

**Figure 4:**
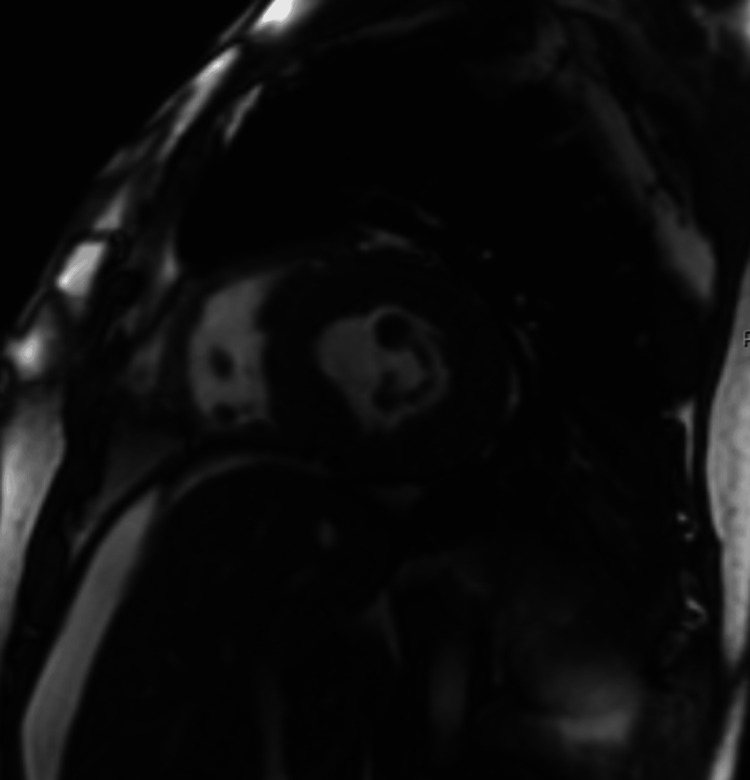
Cardiac MRI with short-axis imaging demonstrating hypertrophy of the left ventricular wall.

**Figure 5:**
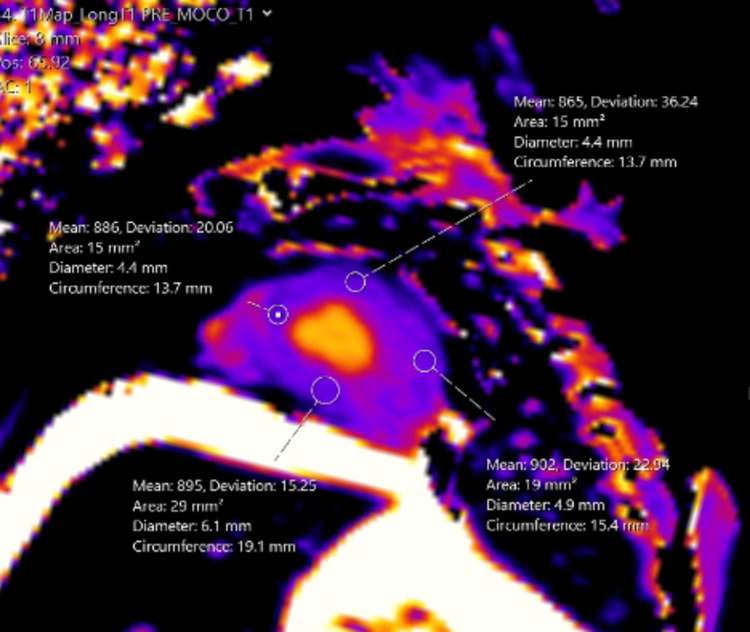
T1 parametric map demonstrating diffuse low T1 values corresponding to the diffuse hyperdensity seen on the CT.

Several months later, the patient presented with acute shortness of breath and hypoxemic respiratory failure secondary to pulmonary edema in ESRD. Echocardiography revealed a decreased ejection fraction from 35-40% to 20%. Myocarditis was suspected; hence, a non-contrast cardiac MRI was performed (Figure 4). T1 maps were conducted to evaluate the myocardium, which incidentally revealed decreased T1 times (Figure 5). On reviewing earlier CTs, it was noted that the patient has diffuse calcifications of her myocardium related to her ESRD. The patient did not have iron overload, Fabry disease, or any other conditions that could account for the low T1 values on cardiac MRI.

## Discussion

Cardiac MRI offers various sequences for differentiating between cardiac abnormalities. Black blood sequences evaluate anatomy and identify mediastinal and pericardial abnormalities; bright blood sequences assess motion, valvular diseases, and flow; and contrast-enhanced sequences provide detailed insights into myocardial conditions [11].

T1 mapping has recently gained popularity. It is a cardiac MRI technique that measures the spin-lattice or longitudinal relaxation time of tissues and displays it on a parametric map. Current T1 mapping techniques refined from the original look-locker method offer greater efficiency and accuracy. The modified Look-Locker inversion recovery (MOLLI) technique, introduced in 2004, uses electrocardiogram (ECG) gating and optimized delays to fit a T1 recovery curve within a 17-heartbeat breath hold. The shortened modified Look-Locker inversion recovery (ShMOLLI), introduced in 2010, further enhanced clinical applicability by reducing breath-hold times, eliminating heart rate dependency, and providing flexibility in estimating a wide range of T1 values, including assessments of edematous tissues and extracellular volume [12,13].

Increased T1 values have been used to describe various pathologies such as cardiac fibrosis, amyloidosis, and myocarditis. Conversely, low T1 values have been associated with other pathologies, such as iron deposition, Fabry disease, and fat deposition. In healthy individuals, normal native T1 time at 1.5T is around 970, with a range of 885-1,073. At 3T, the MOLLI technique has a mean native T1 time at around 1,100, with a range of 964-1,290. However, the T1 times are magnet and institution-specific. At our institution with a 1.5T magnet, our average normal T1 times range from 990 to 1,080. Our patient had a low T1 of 850, which was significantly lower than normal and corresponded to diffuse myocardial calcifications [14,15].

Although rare, myocardial calcification can have severe cardiac consequences. It can lead to increased morbidity and mortality due to chamber dilatation, arrhythmia, and valvular dysfunction [16]. Myocardial calcification can result from various etiologies, with dystrophic calcifications manifesting after myocardial infarction being the most common. Less common causes include metastatic calcifications, which can occur in ESRD and are usually associated with abnormal calcium metabolism [17].

A review of the literature reveals that myocardial calcification has been mentioned only once as a contributor to low T1 values in T1 mapping in a case report by Nijjar and Okasha [18], who described acute myocardial calcifications in a patient with acute myelogenous leukemia. In contrast, ESRD typically results in elevated native T1 values, indicating higher levels of myocardial fibrosis. Our case, however, stands out as an exception, demonstrating low T1 values attributable to calcifications.

## Conclusions

Our case supports the notion that myocardial calcifications can be a potential cause of low T1 values, which can be readily confirmed with a non-contrast chest CT. Additionally, it highlights an exception to the typical pattern in patients with ESRD, who generally exhibit high native T1 values due to myocardial fibrosis.
